# Column Selection for Biomedical Analysis Supported by Column Classification Based on Four Test Parameters

**DOI:** 10.3390/ijms17010136

**Published:** 2016-01-21

**Authors:** Alina Plenis, Natalia Rekowska, Tomasz Bączek

**Affiliations:** Department of Pharmaceutical Chemistry, Medical University of Gdańsk, Hallera 107, 80-416 Gdańsk, Poland; aplenis@gumed.edu.pl (A.P.); nataliarekowska@gumed.edu.pl (N.R.)

**Keywords:** column classification system, Katholieke Universiteit Leuven method, high-performance liquid chromatography, moclobemide and its two metabolites, human plasma, factor analysis

## Abstract

This article focuses on correlating the column classification obtained from the method created at the Katholieke Universiteit Leuven (KUL), with the chromatographic resolution attained in biomedical separation. In the KUL system, each column is described with four parameters, which enables estimation of the *F_KUL_* value characterising similarity of those parameters to the selected reference stationary phase. Thus, a ranking list based on the *F_KUL_* value can be calculated for the chosen reference column, then correlated with the results of the column performance test. In this study, the column performance test was based on analysis of moclobemide and its two metabolites in human plasma by liquid chromatography (LC), using 18 columns. The comparative study was performed using traditional correlation of the *F_KUL_* values with the retention parameters of the analytes describing the column performance test. In order to deepen the comparative assessment of both data sets, factor analysis (FA) was also used. The obtained results indicated that the stationary phase classes, closely related according to the KUL method, yielded comparable separation for the target substances. Therefore, the column ranking system based on the *F_KUL_*-values could be considered supportive in the choice of the appropriate column for biomedical analysis.

## 1. Introduction

The continuously broadened application of reversed-phase high-performance liquid chromatography (RP-HPLC, RPLC) in many various areas, including drug analysis, has expanded the demand for new generations of RPLC stationary phases packing columns of different geometry which would offer better selectivity, efficiency and chemical stability. In effect, several hundred different RPLC columns are commercially available on the market today. However, chromatographic stationary phases often differ in terms of the ligand type and the characteristics of the silica material used as support, as well as in the technique applied to synthesise the packing material [1,2]. Moreover, the polar and ionic features of the RPLC phases responsible for secondary interaction mechanisms often define the unique attributes of the specific RPLC-phase. In consequence, although many of the RPLC stationary phases are nominally identical, their chromatographic performance can differ considerably, making the proper selection of a suitable stationary phase for a particular chromatographic analysis challenging [3,4]. This is the case when e.g., the required column is not available and the analyst needs to find the best alternative. Furthermore, the classification system can be a useful tool when developing a new method. Many papers reporting methods of characterising stationary phases have been published to date to resolve this problem, including the method proposed by Galushko [5], the chromatographic tests reported by Stella *et al.* [6,7], the quantitative structure-retention relationships (QSRRs) delivered by the Kaliszan group [8,9,10,11], the hydrophobic-subtraction model (HSM) published by the Snyder-Dolan group [12,13], and the alternative propositions to the LC column selectivity introduced by Tanaka [14], Euerby [15,16,17,18], Visky [19], Veuthey [20,21,22] and others [23,24,25,26,27]. Another method—a simple chromatographic test procedure of characterising and ranking RPLC C18 columns—has been reported by Hoogmartens and his co-workers from the Katholieke Universiteit Leuven (KUL) [28,29]. In the KUL approach, each column is described against four test parameters: the retention factor of amylbenzene (*k*′*_amb_*) estimating hydrophobicity, the relative retention factor of benzylamine/phenol at pH 2.7 (*rk*′*_ba/ph pH2.7_*) indicating possible silanol activity, the relative retention factor of triphenylene/o-terphenyl (*rk*′*_tri/o-ter_*) describing steric selectivity, and the retention factor of 2,2′-dipyridyl (*k*′*_2_*_,*2′-d*_) reflecting silanol activity and metal impurities [30]. Stage one in the KUL procedure involves choice of four reference parameters corresponding with a freely selected reference column, or of the specific reference column. Then, the *F_KUL_*-values for the tested columns are established according to the formula [31]:

*F_K__UL_ = *(*k*′*_amb_*_, *ref*_ − *k*′*_amb_*_, *i*_)^2^ + (*rk*′*_ba/ph pH2.7_*_, *ref*_ − *rk*′*_ba/ph pH2.7_*_, *i*_)^2^ + (*rk*′*_tri/o-ter_*_, *ref*_ − *rk*′_t*ri/o-ter*, *i*_)^2^ + (*k*′*_2_*_,*2′-d*, *ref*_ − *k*′*_2_*_,*2′-d*, *i*_)^2^ (1)

Finally, the ranking list of the tested columns based on the *F_KUL_*-values is calculated under the rule that the smaller the *F_KUL_*-value, the more similar column *i* is to the reference column. This means that columns with *F_KUL_* < 2 offer the highest probability of selecting the appropriate reference alternative. The probability goes down in the case of stationary phases with 2 < *F_KUL_* < 6, and the lowest value achieved characterises columns treated as low ranking (*F_KUL_* > 6) [31,32].

Of course, any column classification method needs to undergo an important test to verify whether stationary phases with similar parameters will give comparable separations in real pharmaceutical and biomedical applications. In the literature, one can find many reports describing the relationships between column ranking and selectivity in the analysis of different active substances [28,29,30,31,32,33,34,35]. The KUL method was also investigated against other column classification methods [32,36,37,38]. Unfortunately, most papers only concern comparative analyses of the *F_KUL_* parameter values determined for stationary phases against the pharmacopoeial test known as System Suitability Test (SST) or Chromatographic Response Function (CRF), conducted to evaluate the separation of such columns in real pharmaceutical applications [28,29,30,31,32,33,34,35,36,37]. However, both parameters are able to define only experimentally determined overall selectivity of selected compounds. In other words, the two parameters provide a general description of the pharmaceutical separation without demonstrating that the stationary phases classified as comparable by the KUL method actually guarantee analogy between the results of the pharmaceutical analysis and those obtained using the reference column. The KUL test procedure has been evaluated for its usefulness in real pharmaceutical applications based on multidimensional evaluation of experimental data [38,39,40,41], however the KUL approach has been applied to real biomedical analysis involving a biological matrix only once [38]. Therefore, there is a need for further studies to verify reliability of the KUL approach in clinical practice. The studies are important for analysts, because the KUL method offers the advantage of being less complicated and easier to perform while giving results comparable to those obtained under other column classification methods. The latter fact implies that from a practical point of view the KUL method can be attractive to analysts developing new methods, and whenever separation “equivalent” to the original column is required in clinical practice.

In the case study, a new biomedical application—separation of moclobemide (M_0_) and its two metabolites: Ro 12-5637 (M_1_) and Ro 12-8095 (M_2_) (Figure S1) in human plasma samples in accordance with the previously reported HPLC method [42] was chosen as a typical clinical application, and performed on 18 columns previously characterised chromatographically. In the reported method, the Nucleosil 100-5 C18 column (Nuc_C18/125/5) was used, and therefore this particular column was chosen as the reference, and the *F_KUL_*-values were established for the other columns described by various physicochemical parameters. Next, the KUL characteristics of all columns and the column test performance results reflecting the raw retention parameters of M_0_ and its two metabolites, including the retention times (*t_R_*), and resolutions (*R_s_*) of the peaks of interest which, as opposed to the CRF parameter clearly distinguish each real biomedical separation, were evaluated using factor analysis (FA). This chemometric analysis was performed to evaluate whether the column classes, closely related in accordance with the KUL characteristics of their physicochemical properties, demonstrated a similar separation for M_0_ and its two metabolites. For a clearer interpretation of the obtained results, FA was carried out on the same number of columns the number used earlier for classification. Then, the FA results were compared to the results of the principal component analysis (PCA) and hierarchical clustering analysis (HCA) previously reported in the literature [28,38,43].

## 2. Results and Discussion

### 2.1. Column Classification

In the study, 17 brands of stationary phases (and 18 columns), their specifications as described in Table S1, were tested. Each column was described against four chromatographic parameters: *k′_amb_*, *rk′_ba/ph pH2.7_*, *rk′_tri/o-ter_*, and *rk′_2_*_,*2-d*_, all calculated on the basis of the retention data of the test analytes, obtained by strictly defined LC methods (Table S2). This enabled calculation of the *F_KUL_-*values for reference column (Nuc_C18/125/5). The obtained KUL characteristics and the ranking list are summarised in Table 1. The data indicate that the hydrophobicity described by *k*′*_amb_* was highest for Sym_C18, while the lowest value was observed for Nuc_C8. Against the criterion of the *rk*′*_tri/o-ter_* values indicating the steric selectivity, the studied column order was observed to ascend from Inert_C8 to SymShield_C8. When the *rk*′*_ba/ph pH2.7_* parameter was taken into account, silanol activity was found to rise beginning with Sym_C8 and ending with Nuc_C8. As concerns the *k*′*_2_*_,*2′-d*_ parameter reflecting possible silanol activity and metal impurities, the highest value was found for Nuc_C18/250/5, whereas the lowest was noted for SymShield_C8. Moreover, when the Nuc_C18/125/5 column was selected as the reference, only Nuc_C18/250/5 was identified as high ranking. The other twelve columns fell in middle ranking positions. The columns from Inert_C8 to SynMax_RP (as indicated in Table 1) resulted in *F_KUL_* values greater than 6 indicating their physicochemical properties are significantly different. In fact, those columns shared significantly lower *rk′_ba/ph pH2.7_* values, while the *k′_amb_*, *rk′_tri/o-ter_*, and *rk′_2_*_,*2-d*_ parameters varied when compared to the reference.

**Table 1:** The column ranking for the separation of moclobemide, obtained using the *F_KUL_*-values compared against the reference Nuc_C18/125/5 column.

Analytical Column	Column Parameters				*F_KUL_*	The Position in the Ranking List (Column No.)
	*k*′*_amb_*	*rk*′*_tri/o-ter_*	*rk*′*_ba/ph pH2.7_*	*k*′*_2,2′-d_*		
Nuc_C18/125/5	5.09	1.57	0.116	21.07	0.00	1
Nuc_C18/250/5	6.09	1.61	0.102	26.01	1.70	2
SynPol_RP	2.74	1.31	0.088	20.28	2.35	3
Varian_C18	3.20	1.18	0.097	22.62	3.04	4
**NovPack_C18**	**9.42**	**1.39**	**0.108**	**18.21**	**3.97**	**5**
**Nuc_C18/125/10**	**6.78**	**1.24**	**0.104**	**12.36**	**4.50**	**6**
**Nuc_C8**	**1.08**	**1.75**	**0.125**	**19.35**	**4.73**	7
**SynFus_RP**	**6.54**	**1.05**	**0.050**	**17.61**	**4.74**	**8**
Luna_C18 (2)	7.82	1.13	0.087	17.61	4.88	9
**Sym_C8**	**5.55**	**1.08**	**0.029**	**14.63**	**4.95**	**10**
**Aqua_C18**	**8.18**	**1.24**	**0.074**	**15.34**	**5.07**	**11**
**Inert_ODS2**	**9.67**	**1.68**	**0.072**	**19.25**	**5.94**	**12**
NucHD_C18	8.76	1.51	0.053	12.53	5.96	13
**GemNX_C18**	**7.19**	**1.12**	**0.061**	**13.12**	**5.97**	**14**
**Inert_C8**	**4.61**	**1.02**	**0.040**	**13.40**	**6.22**	**15**
**SymShield_C8**	**3.46**	**1.78**	**0.031**	**7.83**	**7.17**	**16**
**Sym_C18**	**10.57**	**1.53**	**0.049**	**15.62**	**9.04**	**17**
**SynMax_RP**	**10.04**	**1.15**	**0.046**	**19.43**	**9.12**	**18**

Meaning of symbols is explained in the text. The columns non-suitable for the separation of the analytes are indicated in bold.

**Figure 1:**
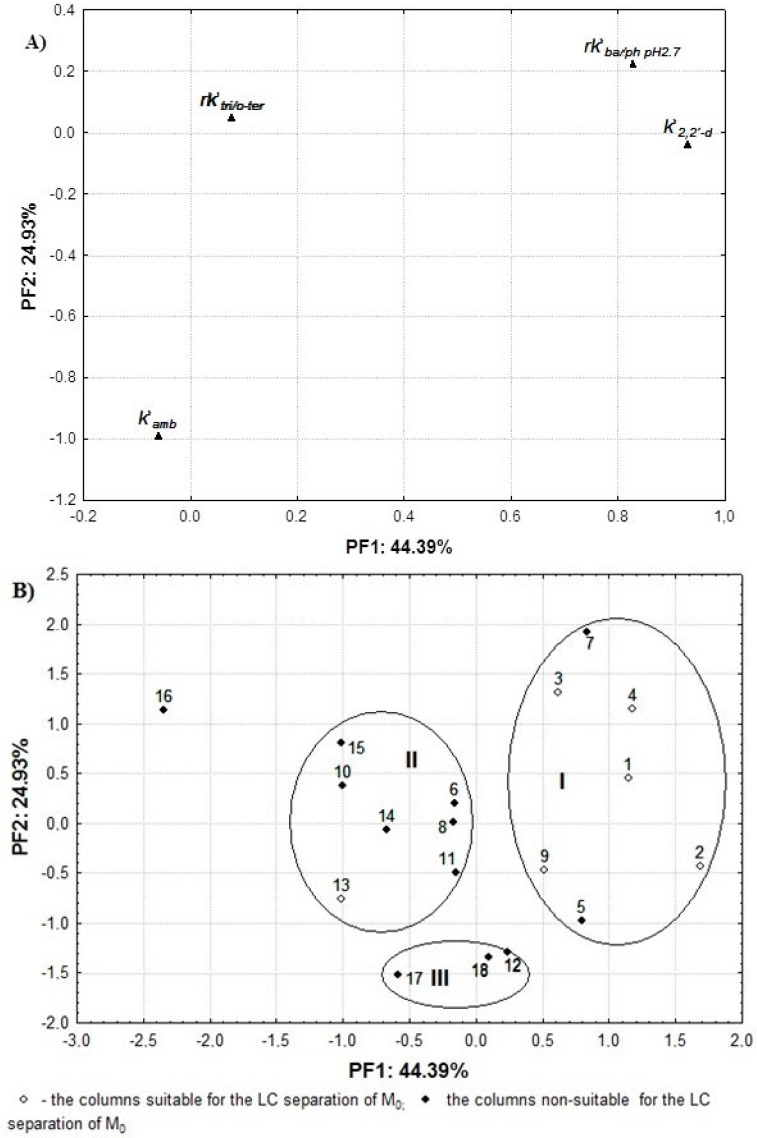
The FA plot of the variables (**A**) and objects (**B**) established based on the auto-scaled KUL test parameters calculated for the 18 columns studied.

Next, an FA based on the *varimax* criterion derived from the auto-scaled KUL results calculated for the 18 brands of columns was performed. This chemometric tool allows for reduction of the number of variables and the detection of the structural relationships between the variables and objects without the loss of an essential information [44]. Thus, an FA enables a more detailed interpretation of the column classification results. Surprisingly, the same stationary phase packing two columns of different lengths lead to different values of *k′_amb_* and *k′_22′-d_*, which seems anormal. Consequently the two *F_KUL_* values for both the same stationary phase and columns 1 and 2 (see Table 1) are different. This could probably due to different column-to-column and batch-to-batch reproducibility of this chromatographic support. For the purposes of the analysis, the numbering of the stationary phases as reported in Table 1 was retained. The two-dimensional FA plots for the variables and objects are illustrated in Figure 1A,B, respectively.

Notably, the 44.39% data variability explained by the first principal factor (PF1), was caused primarily by the variability of *rk′_2_*_,*2′-d*_ and *rk′_ba/ph pH2.7_*. The two variables were located, close to each other, on the upper right side of the plot (Figure 1A). It confirms that silanol activity (*rk′_2_*_,*2′-d*_ and *rk′_ba/ph pH2.7_*) and metal impurities (*rk′_2_*_,*2′-d*_) are of predominant influence on differentiation of the stationary phases based on KUL classification results. In fact, all stationary phases studied, except for column SymShield_C8, shared high *rk′_2_*_,*2′-d*_ parameters (>12), though their values differed (Table 1). As concerns *rk′_ba/ph pH2_*, various low (<0.1) and intermediate (<0.3) parameter values were obtained in calculations. Hence, those stationary phases demonstrate low or intermediate silanol activity.

The *k′_amb_* and *rk′_tri/o-ter_* parameters were found positioned at the bottom and on the upper left side of the graph, where the variability of *k′_amb_* was explained mainly by the PF2. In other words, the FA results showed that steric selectivity of the stationary phases estimated by the *rk′_tri/o-ter_* parameter did not differentiate the columns studied to any significant extent. In fact, according to Table 1, relatively low values of these parameters (<1.7) were calculated for 16 tested stationary phases. Only Nuc_C8 and SymShield_C8 proved having higher steric selectivity (*rk′_tri/o-ter_* > 1.7). In summary, the first two PFs together account for 69.32% of the total variance of the original data set. The data corroborate the previously reported literature data, where the positions of the variables on the PCA plot were comparable to those observed in the FA plot [28].

The obtained classification data are consistent with the data reported in Ref. [43] where the stationary phases, except for Nuc_C18/250/5, were classified by the QSRR models supported by two chemometric tools, namely the principal component analysis (PCA) and hierarchical clustering analysis (HCA). The PCA results based on the QSRR characteristics, including the 17 columns tested in this investigation, also showed that those seventeen stationary phases were respectively distributed in the same three clusters, except for column 11 which was out of cluster II in the PC analysis. Among them, eight stationary phases (Nuc_C18/125/5; SynPol_RP; NovPack_C18; SynFus_RP; Luna_C18; Sym_C8; Inert_ODS2; SynMax_RP) were found in the same positions on the PCA and FA plots built by the QSRR and the KUL methods, respectively. In other words, both approaches to the selection of a column resulted in a similar classification, even if the statistical methods used were different (FA and PCA). The data further indicated that correspondence between the KUL and QSRR characteristics was higher than that obtained for QSRR and other column classification systems, previously described in the literature [32,36,37,38]. On the other hand, it is interesting whether the probability of suitable stationary phase selection in clinical practice is comparable to the probability of the QSRR models.

### 2.2. Column Selectivity in Analysis of Moclobemide

As mentioned above, the verification of the theoretical KUL results in biomedical analysis for 18 columns was based on the measurement of M_0_ and its two metabolites—Ro-1256 (M_1_) and Ro 12-8095 (M_2_) in human plasma, performed according to the previously reported LC method [42]. In the assay, the same chromatographic conditions were applied for all examined columns in the analysis of the Quality Control samples (QCs) and of the real samples collected from healthy volunteers after single application of M_0_. Moreover, separation for all analytes was assessed against the main criterion of peak resolutions Rs ≥ 1.5. The obtained retention data confirmed that the *t_R_* values of M_0_ fell in the range of 1.50–7.95 min, and the last detected compound (I.S.) was recorded in between 3.08 and 16.53 min (Table 2).

Notably, the highest ranked column, Nuc_C18/250/5, and two stationary phases of *F_KUL_* < 4 were suitable for the LC separation of M_0_ and its two metabolites (3/4—75%) (Table 1). As concerns the stationary phases with the *F_KUL_*-values falling between 4 and 6, only two columns (No. 9 and 13) yielded proper LC analysis of the compounds of interest (2/9—22.2%), whereas no columns in the lower ranking positions (*F_KUL_* > 6) were suitable for the analysis (0/4—0%). This is consistent with the commonly accepted rule that the probability of choosing the proper column decreases with the increasing *F_KUL_*-values. Notably, all columns suitable for the LC analysis of the analytes, except for NucHD_C18, were characterised by high values of the *rk*′*_ba/ph pH2.7_* and *rk′_2_*_,*2-d*_ parameters. This indicates that higher silanol activity and metal impurities increase the probability of proper LC separation. The observation may be related with the fact that all compounds of interest have atoms of oxygen and nitrogen in their structure, able to create dipole-dipole or dipole-dipole induced interactions with the stationary phase (Figure S1).

**Table 2:** Summary of data set of *t_R_* and *R_s_* for M_0_, its two metabolites and I.S. in column performance test for eighteen columns studied.

Substances:		M_0_	M_1_		M_2_		Phenacetin (I.S.)	
No.	Analytical Column:	*t_R_*	*t_R_*	*R_s_*	*t_R_*	*R_s_*	*t_R_*	*R_s_*
1	Nuc_C18/125/5	2.51	2.95	1.61	5.15	8.39	5.53	1.64
2	Nuc_C18/250/5	3.95	4.56	2.56	7.31	8.44	8.75	3.25
3	SynPol_RP	3.07	3.46	2.74	7.40	14.81	8.43	3.31
4	Varian_C18	7.95	9.35	1.82	10.33	1.58	11.95	2.02
**5**	**NovPack_C18**	**3.46**	**4.05**	**1.71**	**7.16**	**5.42**	**7.83**	**0.83**
**6**	**Nuc_C18/125/10**	**1.50**	**1.63**	**1.13**	**3.08**	**4.83**	**3.08**	**0.00**
**7**	**Nuc_C8**	**3.96**	**4.81**	**1.97**	**11.08**	**8.48**	**11.08**	**0.00**
**8**	**SynFus_RP**	**4.03**	**4.03**	**0.00**	**14.13**	**31.43**	**16.53**	**3.98**
9	Luna_C18 (2)	1.61	2.28	5.54	8.15	26.91	9.56	4.09
**10**	**Sym_C8**	**3.80**	**3.80**	**0.00**	**12.90**	**24.42**	**15.03**	**4.96**
**11**	**Aqua_C18**	**4.55**	**5.15**	**0.78**	**13.61**	**11.30**	**15.41**	**1.91**
**12**	**Inert_ODS2**	**2.20**	**2.20**	**0.00**	**7.43**	**18.84**	**8.76**	**3.32**
13	NucHD_C18	3.45	3.95	2.60	8.13	14.24	9.48	3.41
**14**	**GemNX_C18**	**2.06**	**2.06**	**0.00**	**5.73**	**17.20**	**7.00**	**4.03**
**15**	**Inert_C8**	**3.90**	**3.90**	**0.00**	**11.71**	**18.86**	**13.68**	**4.24**
**16**	**SymShield_C8**	**3.75**	**3.75**	**0.00**	**11.16**	**14.13**	**14.23**	**3.27**
**17**	**Sym_C18**	**3.33**	**3.33**	**0.00**	**11.10**	**29.63**	**13.06**	**4.05**
**18**	**SynMax_RP**	**2.21**	**2.21**	**0.00**	**6.83**	**23.68**	**8.73**	**5.89**

Meaning of symbols is explained in the text. The columns non-suitable for the LC separation of the analytes are indicated in bold.

**Figure 2:**
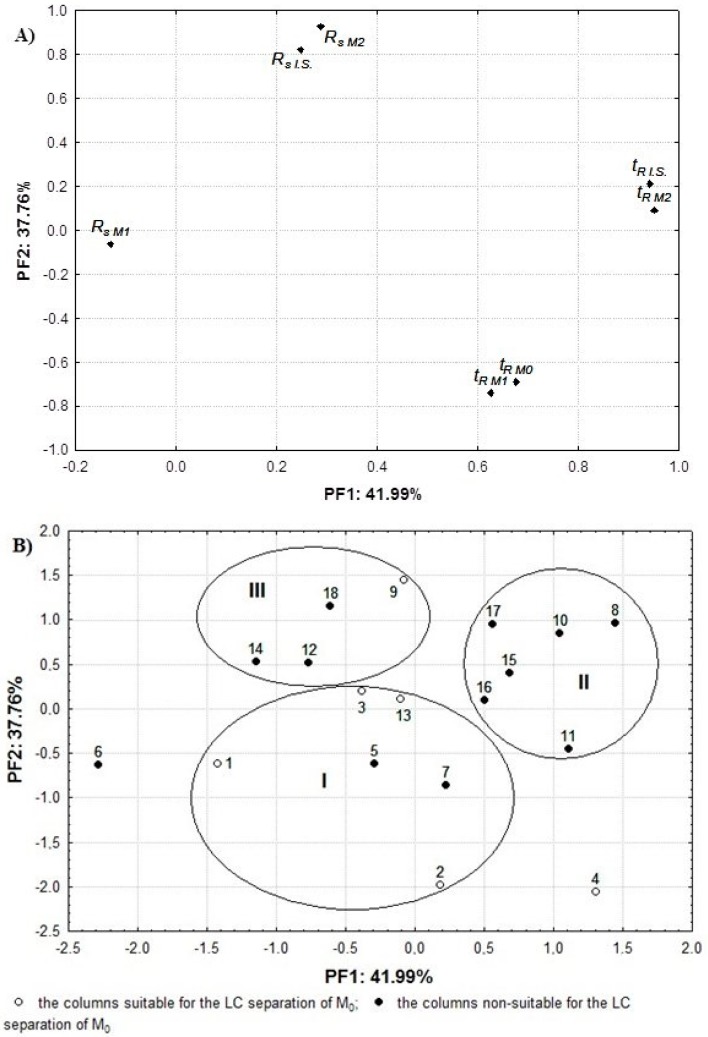
The FA plot for the variables (**A**) and objects (**B**) established for the auto-scaled retention parameters (*t_R_* and *R_s_*) of the compounds of interest during the column performance test based on the LC analysis of M_0_ and its two metabolites.

Next, an FA derived from the auto-scaled column test performance results was performed for the 18 columns studied. The FA plots for the variables and objects are shown in Figure 2A,B, respectively.

It should be emphasised that the variance of the *t_R M2_* and *t_R I.S_*_._, and *t_R M0_* variables was mainly due to the PF1. The *t_R M2_* and *t_R I.S._* variables were located, close to each other, in the right part of the middle section of the graph, while *t_R M0_* and the *t_R M1_* parameter were found on the bottom side of the plot. The variability of *R_s M2_*, *R_s I.S._*, both positioned on the upper side of the graph, and that of the *t_R M1_* was explained mainly by the PF2. Thus, the first two PFs explained 79.75% of the data variability (Figure 2A). When the FA plot was compared to the loading of the PC plot reported in Reference [43] as Figure 4A, it could be noticed that the location of the variables was similar even though the variability of the *t_R M1_* parameter was mainly explained by PF2, whereas in the PCA it was related to PC1.

Figure 2B shows the FA plot for the objects where almost all columns were positioned in three clusters. Only two columns (No. 4 and 6) were found to be outliers on opposite sides of cluster I. It should also be noted that the locations of the columns studied on the FA plot were generally well correlated with the *F_KUL_-*values established by the KUL procedure (Table 1). Hence, columns No. 1–3, 5, 7, and 13 were positioned in cluster I. For these, the intermediate values of *t_R M0_* and the higher *R_s_* of the M1 parameters were calculated. Ultimately, four columns (No. 1–3 and 13) were identified as suitable for an appropriate LC analysis of the target compounds. The columns located in cluster II were characterised by insufficient *R_s_* of the M_1_, despite long-time analysis (Table 2). Consequently, none of the columns can be used for analysis of M_0_ and its metabolites. The columns in cluster III (Figure 2B) offered a shorter and intermediate *t_R_* of the target compounds, while their *R_s_* parameters for M_0_, M_2_ and I.S. were intermediate or higher (Table 2). Nevertheless, the *R_s_* of M_1_ < 1.5 were calculated for column No. 12, 14, and 18. Concluding, only column No. 9 of the phases in group III was suitable for separation of the analytes in human plasma (Figure 2B). Moreover, as mentioned above, two brand columns were found to be outliers. Column No. 4, described by the highest values of *t_R M0_* and *t_R M1_* and a high *R_s M1_* parameter, ensured appropriate separation of the target compounds (Table 2). In contrast, the lowest *t_R_* of the analytes was calculated for column No. 6. Unfortunately, because of insufficient separation of M1 and the I.S., this stationary phase proved unsuitable for the analysis of M_0_ and its metabolites in human plasma.

Notably, most columns were located in the same positions on both the FA plot based on four column parameters (Figure 1B), and on the FA plot derived from the column test performance data (Figure 2B). Therefore, almost all columns from cluster I (5/6) were also found in cluster I, as presented in Figure 1B, while the column No. 8, 10, 11, and 15 were positioned in cluster II. On the other hand, some columns, namely 4, 6, 9, 13, 14, 16 and 17, were found in different positions than on Figure 1B. Among them, columns 4, 6, 9 and 13 were also incorrectly classified by the QSRR models [43], where 24 stationary phases were tested, including the 17 stationary phases tested in this investigation. The differences can, to an extent, be explained by the fact that the column temperature used in the KUL and QSRR procedures was higher than in the column performance test, which might have affected the chromatographic behaviour of the analytes [45,46].

Nevertheless, it is worth emphasising that five column classes, closely linked by the KUL method and falling in cluster I (Figure 1B), ensured suitable separation of M_0_ and its metabolites in human plasma. Therefore, the KUL procedure proved capable of increasing the probability of appropriate selection of the column from the initial value of 33.3% (6/18—Table 1) to 71.42% (5/7—cluster I, Figure 1B). Moreover, verification of the test results confirmed that the application of the KUL method yielded comparable results to those of the QSRR approach. In a previous study [43], the probability of choosing the suitable column increased from the initial value of 37.5% to 62.5% when using the QSRR models, or to 66.6% in accordance with the PCA and HCA. Hence, the present study confirms the usefulness of the KUL method in the clinical practice involving analysis of more complicated biological matrices. Moreover, the probability of selecting the appropriate column is slightly higher when the traditional way of comparing the *F_KUL_* values, rather than an FA assay, is employed. On the other hand, graphical visualisation of the experimental data sets under the FA enables more accurate interpretation of the KUL classification and the column test performance results. The results can be of interest to the analysts in both their cognitive and practical aspects, because the KUL procedure is less complicated and easier to perform than other column classification systems.

## 3. Experimental Section

### 3.1. Column Examination

In this study, the eighteen investigated RPLC columns were delivered by the manufacturers or distributors. Their characteristics are given in Table S1.

### 3.2. Chemicals

The test substances used in the KUL method, namely uracil, *o-*terphenyl, triphenylene, benzylamine, amylbenzene (*n*-pentylbenzene), and 2,2′-dipyridyl, were purchased from Sigma-Aldrich (St. Louis, MO, USA), whereas phenol was delivered by POCH (Gliwice, Poland). For preparation of the mobile phases, the HPLC grade of methanol was obtained from Merck (Darmstadt, Germany), while potassium dihydrogen phosphate and 85% *ortho*-phosphoric acid, both reagents of the analytical-reagent grade, were delivered by Sigma-Aldrich (St. Louis, MO, USA). All reagents were used as received without further purification. Water was pre-treated in a Milli-Q Water Purification System (Millipore Corporation, Bedford, MA, USA).

Moclobemide (M_0_) used in the column performance test was purchased from Biovena Pharma (Warsaw, Poland), whereas its two metabolites: Ro 12-5637 (M_1_) and Ro 12-8095 (M_2_) were supplied by Hoffmann-La Roche Ltd. (Basel, Switzerland). Phenacetin, applied as the internal standard (I.S.), was donated by Sigma-Aldrich (St. Louis, MO, USA). Acetonitrile and dichloromethane, both solvents of HPLC grade, were obtained from Merck (Darmstadt, Germany), while sodium hydroxide was delivered by POCH (Gliwice, Poland). The control plasma samples were obtained from healthy volunteers.

### 3.3. Equipment and LC Conditions

All LC determinations were carried out on an ACME 9000 system (Younglin Instrument Corporation, Anyang, the Republic of Korea), containing of a pump (SP 930D), thermostat (CTS30), auto-sampler equipped with a 20 μL loop, and a 730D UV/VIS detector. The AutoChro-3000 Chromatography Data System was applied for data acquisition.

In order to classify the columns using the KUL procedure, three isocratic chromatographic methods were applied to the selected analytes, as shown in Table S2. In each method, the column temperature of 40 °C and the flow rate of 1 mL/min were used while the UV detector was set at 254 nm.

The LC analysis of M_0_ and its two metabolites in human plasma was carried out using a mixture of acetonitrile and water adjusted to pH 2.7 with 85% *ortho*-phosphoric acid (25:75, *v*/*v*) as the mobile phase, at the flow rate of 1 mL/min. The analytes were determined with the UV detector set at 239 nm, and the LC system was maintained at the room temperature.

### 3.4. Column Classification

To classify the columns, three chromatographic methods were used in a defined order (A-B-C) (Table S2). The relative retention factor benzylamine/phenol at pH 2.7 (*rk*′*_ba/ph pH2.7_*) in method A, the retention factor of 2.2′-dipyridyl (*k*′*_2_*_,*2′-d*_) in method B, the retention factor of amylbenzene (*k*′*_amb_*), and the relative retention factor triphenylene/*o*-terphenyl (*rk*′_t*ri/o-ter*_) in method C were established according in Equations presented in Table S2 using the dead time calculated in method C employing uracil. All determinations were done three times resulting in the RSD values below 1%. Based on the obtained results, the KUL characteristics of all examined columns containing the four chromatographic parameters were calculated. Next, upon choosing the Nuc_18/125/5 stationary phase as the reference, the *F_KUL_*-values for the other columns were established according to Equation (1). For this purpose, the software available on-line at http://pharm.kuleuvan.be/pharmchem/Pages/ccs.html was used, and all examined columns were numbered according to their position on the ranking list. In addition, factor analysis (FA) was performed using the Statistica 12.0 package (StatSoft, Tulsa, OK, USA) based on the auto-scaled values of four column parameters, established for all columns.

### 3.5. Column Test Performance

The practical test of the KUL method for biomedical analysis was performed on 18 RPLC columns during the separation of moclobemide (M_0_) and its two metabolites: Ro 12-5637 (M_1_) and Ro 12-8095 (M_2_) in human plasma samples prepared according to the sample preparation procedure prescribed in Reference [42]. Both the QCs and real human plasma samples from healthy volunteers collected after administration of a 150 mg dose of M_0_ were treated in the same manner. In brief, phenacetin at the concentration of 800 ng/mL was added to 1 mL of the human plasma sample to serve as the internal standard (I.S.). Moreover, while preparing the QCs containing the target compounds at low, middle, and high concentration levels appropriate volumes of the working standard solutions of the analytes at a concentration of 10 μg/mL were added to 1 mL of the human plasma sample to achieve 100, 800, and 1500 ng/mL for M_0_; 60, 100, and 150 ng/mL for M_1_; and 50, 500, and 1000 ng/mL for M_2_, respectively. Next, the sample was mixed with 4 mL of dichloromethane and 200 μL of 1 M NaOH, mechanically shaken for 10 min and centrifuged for 15 min (1000 *g*). Then, dichloromethane was transferred to a clean test tube and the solvent evaporated to dryness in a water bath at 45 °C under an air stream. Finally, the residue was reconstituted in 200 μL of acetonitrile-water mixture (3:2, *v*/*v*), centrifuged at 8000× *g* for 5 min, and 20 μL of the aliquot was injected into the LC system.

The study began with the LC analysis of M_0_ and its two metabolites, performed on the Nuc_C18/125/5 column. Then, the other examined stationary phases were applied to analyse the target compounds in the same chromatographic conditions as those reported in Section 3.3, and the retention parameters, namely *t_R_* and *R_s_* of the peaks of interest for M_0_, M_1_, M_2_ and the I.S., were evaluated for all columns studied. Finally, an FA based on the auto-scaled column test performance results established for the 18 columns studied was performed.

## 4. Conclusions

In the study, LC columns were classified based on the KUL method and their selectivity toward moclobemide and its two metabolites in human plasma, using 18 RPLC columns. For the evaluation of similarities and differences between the column classification system and the column test performance, the traditional approach of comparing the calculated *F_KUL_*-values was adopted alongside a factor analysis (FA) using the *varimax* algorithm as an interesting alternative to the principal component analysis (PCA) and hierarchical clustering analysis (HCA). In general, both the traditional and the multivariate approach based on the FA enabled more appropriate column selection. Moreover, the *F_KUL_*-values obtained under the KUL method and the localisations of the columns studied on the FA plots, were significantly correlated. The FA results also indicated that the column classes, closely related according to the KUL method, offered comparable separation of the analytes. The fact confirms that the KUL classification method yields results which allow for the selection of columns that will be similar to or dissimilar from the reference column at a relatively high certainty level. Thus, the column ranking system based on four column parameters could be considered supportive in the choosing of the appropriate column for the specific biomedical application.

## Supplementary Materials

Supplementary materials can be found at http://www.mdpi.com/1422-0067/17/1/136/s1.
