# Supplementary material for: Column Selection for Biomedical Analysis Supported by Column Classification Based on Four Test Parameters
Source: Int J Mol Sci. 2016 Jan 21;17(1):136. doi: 10.3390/ijms17010136 (PMC4730375; doi:10.3390/ijms17010136)
Supplement: Supplementary file 1 [file ijms-17-00136-s001.pdf]

Supplementary Materials: Column Selection for Biomedical Analysis Supported by Column Classification Based on Four Test Parameters

Alina Plenis, Natalia Rekowska and Tomasz Bączek

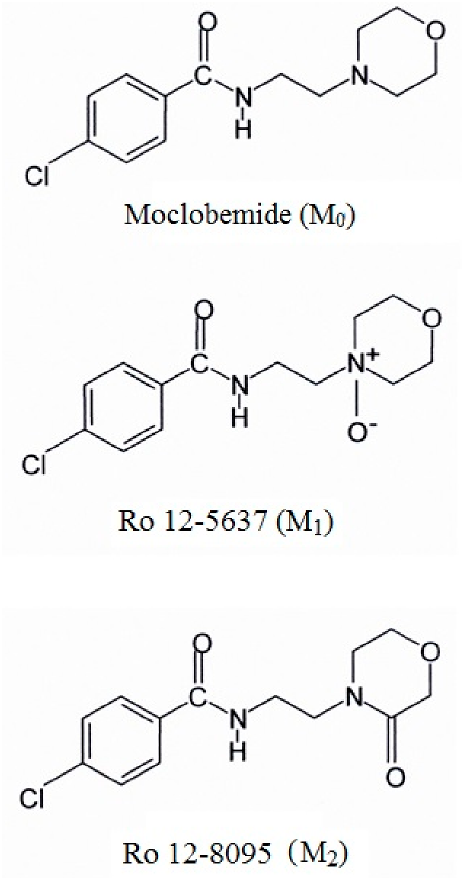

Figure S1. Chemical structures of moclobemide (M_0) and its two metabolites: Ro 12-5637 (M_1) and Ro 12-8095 (M_2).

Table S1. Specifications for the examined columns as provided by the manufacturer.

Name of the Column	Length (mm)	Internal Diameter (mm)	Particle Size (µm)	Modification Packed Material by the Groups:	Carbon Load (%)	Pore Size (Å)	Surface Area ($m^2 \cdot g^{-1}$)	Silica	Endcap.	Manufacturer/Supplier	Abbreviation
Nucleosil 100-5 C18	125	4.0	5	C18	15	100	350	A	+	Knauer	Nuc_C18/125/5
Nucleosil 100-5 C18	250	4.0	5	C18	15	100	350	A	+	Knauer	Nuc_C18/250/5
Synergi Polar-RP	150	4.6	4	ether-linked phenyl	11	80	475	B	+ Polar	Phenomenex	SynPol_RP
Varian Pursuit C18	150	4.6	5	C18	12.9	200	200	B	+	Varian	Varian_C18
Nova-Pack C18	150	3.9	4	C18	7.3	60	120	A	+	Waters	NovPack_C18
Nucleosil 100-10 C18	125	4.0	10	C18	15	100	350	A	+	Knauer	Nuc_C18/125/10
Nucleosil 100-7 C8	250	4.0	7	C8	8.5	100	350	A	−	Macherey-Nagel	Nuc_C8
Synergi Fusion- RP	250	4.6	4	polar embedded C18	12	80	475	B	+	Phenomenex	SynFus_RP
Luna C18 (2)	150	4.6	3	C18	17.5	100	400	B	+	Phenomenex	Luna_C18
Symmetry C8	250	4.6	5	C8	11.7	100	335	B	+	Waters	Sym_C8
Aqua C18	250	4.6	5	C18	15	125	320	B	+ Polar	Phenomenex	Aqua_C18
Inertsil ODS2	150	4.6	5	C18	18.5	150	320	B	+	Hichrom	Inert_ODS2
Nucleosil 100-5 C18 HD	250	4.0	5	C18	20	100	350	B	+	Macherey-Nagel	NucHD_C18
Gemini-NX C18	150	4.6	5	C18	14	110	375	B	+ Hybrid	Phenomenex	GemNX_C18
Inertsil C8	250	4.6	5	C8	11	150	320	B	+	MZ-Analysentechnik GmbH	Inert_C8
Symmetry Shield RP8	250	4.6	5	polar embedded C8	15	100	335	B	+	Waters	SymShield_C8
Symmetry C18	250	4.6	5	C18	19	100	335	B	+	Waters	Sym_C18
Synergi-Max-RP	150	4.6	4	C12	17	80	475	B	+	Phenomenex	SynMax_RP

Table S2. The chromatographic test methods and the samples analysed in the KUL approach for testing stationary phases.

Method	Mobile Phase	Sample	Column Parameter	Equations
A	Methanol-water-0.2 M KH_2PO_4 at pH 2.7 [a] (34:90:10, *w*/*w*/*w*)	Benzylamine (*ba*)<br>Phenol (*ph*)	$rk'_{ba/ph\ pH\ 2.7}$	$rk'_{ba/pH\ 2.7} = \frac{t_{R_{ba}} - t_{R_u}}{t_{R_{ph}} - t_{R_u}}$
B	Methanol-water-0.2 M KH_2PO_4 at pH 6.5 [a] (34:90:10, *w*/*w*/*w*) [a]	2,2′- Dipyridyl	$k'_{2,2'-d}$	$k'_{2,2'-d} = \frac{t_{R_{2,2'-d}} - t_{R_u}}{t_{R_u}}$
C	Methanol-water (317:100, *w*/*w*)	Uracil (*u*)<br>Amylbenzene (*amb*)<br>*o*-Terphenyl (*o-ter*)<br>Triphenylene (*tri*)	k'_{amb}, $rk'_{tri/o-ter}$	$k'_{amb} = \frac{t_{R_{amb}} - t_{R_u}}{t_{R_u}}$<br>$rk'_{tri/o-ter} = \frac{t_{R_{tri}} - t_{R_u}}{t_{R_{o-ter}} - t_{R_u}}$

Method	Sample composition
A	5 mg of benzylamine and 5 mg of phenol in 10 mL of mobile phase A
B	3 mg of 2,2′-dipyridyl in 10 mL of mobile phase B
C	0.1 mg of uracil, 7 mg of amylbenzene, 0.2 mg of *o*-terphenyl and 0.02 mg of triphenylene in 10 mL of mobile phase C

[a] The pH adjustments were performed before adding the organic compound of the mixture [32].
